# From security to spending: how China’s long-term care insurance pilot drives urban household consumption

**DOI:** 10.3389/fpubh.2025.1667168

**Published:** 2025-11-14

**Authors:** Tao Li, Linlin Wang

**Affiliations:** School of Economics, Qingdao University, Qingdao, Shandong, China

**Keywords:** long-term care insurance, urban household consumption, social healthcare service provision, urban innovation capacity, labour wage levels

## Abstract

**Introduction:**

As China confronts rapid population ageing and rising demand for older people’s care, the establishment of a comprehensive long-term care insurance system has become an important institutional innovation. This study explores whether and how the implementation of the long-term care insurance pilot policy affects urban household consumption, a key component of domestic demand.

**Methods:**

This study employs a Difference-in-Differences approach based on panel data from 232 prefecture-level and above cities in China between 2011 and 2018 to identify the causal effect of the long-term care insurance pilot. The analysis further investigates the underlying mechanisms, including the expansion of public healthcare services, the enhancement of urban innovation capacity, and the improvement of wage levels. It also examines heterogeneity across different city types and spatial spillover effects.

**Results:**

Empirical evidence suggests that the implementation of the long-term care insurance pilot has a statistically significant positive impact on urban residents’ consumption, increasing it by 7.35 per cent, equivalent to approximately 206.14 yuan. This effect is primarily driven by the reduction in informal care burdens, the rise in labour market participation, improved accessibility of medical services, and the enhanced application of technology in the care industry. The impact is more pronounced in cities with net population inflows and broader pilot coverage. Specifically, the introduction of the long-term care insurance raises urban residents’ consumption by 8.22 per cent and 8.44 per cent in these cities, corresponding to 240.82 yuan and 236.68 yuan respectively, whereas such effects are not evident in cities with population outflows or narrower pilot coverage. Furthermore, the policy exhibits a notable spatial spillover effect, increasing the consumption levels of neighbouring non-pilot cities by up to 9.86 per cent, approximately 275.16 yuan.

**Discussion:**

These findings highlight the economic significance of long-term care system reform. Beyond meeting the care needs of an ageing population, the long-term care insurance pilot serves as a policy tool to stimulate consumption, promote employment, and narrow regional disparities. The spatial spillover effects suggest that scaling up the program may generate broader regional benefits, providing valuable insights for the further development of China’s care system and social security reform.

## 1 Introduction

Keynesian consumption theory emphasises the important role of consumer spending in promoting economic growth (1). As a crucial macro indicator of economic dynamics, changes in consumer spending not only reflect variations in residents’ income levels and expectations, but also have a profound effect on the pattern of economic growth and aggregate demand. In mature economies, the domestic demand-led growth model frequently relies on stable and sustained consumer spending (2). Conversely, in emerging economies, the expansion of consumer spending is a crucial factor in optimising the economic structure and promoting industrial upgrading (3). However, rising trade protectionism, deglobalisation, and volatile international political conditions have led to weak growth in consumer spending as global consumers face multiple risks to their economies, security, and health (4–6). Concurrently, the global economic transformation following COVID-19 has worsened income inequality worldwide (7). This phenomenon has raised the financial vulnerability of non-high-income households, increasing their concerns about financial risk and thus hampering the growth of consumer spending (8). Long-term care insurance (LTCI) may play a role in reducing the financial pressure on households and increasing their purchasing power. As part of the social security system, LTCI seeks to offer financial assistance to disabled workers while mitigating the financial risks their families face due to long-term care costs (9). Given rising income inequality, increasing the coverage and level of protection of LTCI may reduce the financial insecurity of low-income groups and hence influence their household consumption decisions.

Due to Confucian cultural characteristics, China and other Northeast Asian countries have long faced low consumer spending, high savings, and insufficient domestic demand to drive economic growth (10, 11). In 2022, final consumption expenditure as a share of GDP was 68% in the United States and 60.6% in India, despite a 0.6 percentage point decline compared to 2021. In contrast, China’s consumer spending as a percentage of GDP is only 39.6% in 2023, much lower than even Japan’s 55%, a country that also belongs to the Confucian cultural sphere^1^. Consumption by urban residents is an important component of consumption, and both theoretical and empirical evidence have confirmed the intrinsic link between urbanisation and changes in consumption demand (12, 13). Following the reform and opening up, China has experienced rapid urbanisation at an average rate of 1% per year (14), and by 2022, the urbanisation rate reaching 65.22% by 2022, but the share of urban household final consumption in GDP is only 38.8%^2^. This indicates that the consumption power of China’s urban residents has not been fully unleashed, and the mismatch between the pace of urban development and the level of consumption expenditure growth is becoming more pronounced. The decrease in consumer demand directly results in lower overall demand, which in turn reduces businesses’ incentives to produce and invest. This phenomenon forms a vicious cycle of demand contraction and supply adjustment, and even generates the risk of deflation (15). Consequently, ensuring steady consumer demand and reinforcing household confidence in expenditure are essential for fostering sustainable development and optimising the economic framework.

Studies have demonstrated that social insurance, such as medical insurance, has been shown to reduce precautionary savings and increase consumption (16). Implementing the LTCI system in urban China can reduce the financial risks of incapacity for individuals, promoting a more stable and predictable socioeconomic environment. This, in turn, enhances consumer spending and thus promotes overall economic activity (17, 18). Practical experience in East Asian countries, such as Japan and South Korea, shows that LTCI, by partially covering care services and costs for the disabled, can alleviate families’ concerns about the uncertainty of future, reduce families’ motivation for precautionary savings, and ultimately increase consumption levels (19). In order to address the issue of disability, China has been implementing pilot policies for a national LTCI system since 2016. In July 2016, Qingdao, Chengde, and 13 other cities were selected as national LTCI pilot sites. The pilot programs have achieved notable success, providing valuable experience for the promotion of the LTCI system (20). Building on the successful outcomes of the initial pilot phase, the LTCI program was further expanded in September 2020 to include an additional 14 cities (21). The implementation of long-term policy pilots serves as a valuable research framework for the analysis of the economic implications of LTCI. Therefore, exploring the potential impact of China’s LTCI system on consumer spending can help understand the role of LTCI policies in the macroeconomy and provide a more comprehensive perspective on China’s shift to a consumption-driven economic growth engine. Furthermore, as the largest developing country in the world, research on the implementation of the LTCI system in China will provide a new perspective for the improvement of the relevant system in developed countries, as well as past experience for the design of the relevant social security system in developing countries.

The present study raises the following research questions: (1) In the context of China’s pilot LTCI system, do household consumer spending differ between pilot and non-pilot regions? In other words, does the LTCI pilot policy effectively affect consumption expenditure? (2) Which cities demonstrated the largest growth in household consumption expenditure after implementing the pilot LTCI system? In other words, for which LTCI pilot cities were the policy more effective? (3) Through which channels do LTCI affect household consumption levels? To answer these questions, this study utilises a quasi-natural experiment to assess how LTCI influences urban household consumption expenditure, conducting both theoretical and empirical mechanism analyses. We provide policy insights for LTCI, with the objective of assisting China in boosting consumer spending and expanding domestic demand in all aspects. This study makes several potential contributions: First, it offers empirical evidence for the efficacy of LTCI on urban households’ consumption expenditures. It combines research on the LTCI system and household consumption expenditure, expanding the scope of studies in these areas and related fields. Second, it elucidates the internal influence mechanisms of LTCI on urban households’ consumption expenditures from the perspectives of social healthcare service provision, urban innovation capacity and labour wage levels, extending the existing influence mechanism, unveiling a new influence mechanism. Finally, it also demonstrates that LTCI has spatial spillover effects on urban household consumption expenditure. The results provide a solid theoretical foundation for the systematic implementation of the LTCI system, the development of China’s care market, the enhancement of urban households’ well-being, the expansion of domestic demand, and the stimulation of consumption.

The remainder of this paper is organised as follows. Section 2 presents the policy background and literature review; Section 3 outlines the theoretical analysis and research hypotheses; Section 4 describes the research design, baseline regression, and robustness checks; Section 5 conducts the heterogeneity analysis; Section 6 discusses the underlying mechanisms; Section 7 provides an extended analysis; and Section 8 concludes with research findings and policy implications.

## 2 Research background

### 2.1 Policy background

From a developmental perspective, the LTCI has progressed from an initial institutional inception to an expansion and deepening of its coverage and to a coordinated advancement. In 2016, the Ministry of Human Resources and Social Security promulgated *the Guiding Opinions on the Launching of Pilot Programmes for the Long-Term Care Insurance System*. Fifteen cities, including Qingdao and Chengde, were selected as national pilot cities for LTCI. This initiative represented the inaugural institutional-level pilot programme for LTCI, with a focus on the exploration of funding mechanisms and benefit payment systems to ensure the financial security of individuals with disabilities. The initial establishment of the LTCI scheme was primarily driven by the escalating care requirements of the disabled population within the context of an ageing society. In the same year, *“Healthy China 2030” Planning Outline* proposed the establishment of a multi-tiered long-term care system, with the aim of encouraging private capital to enter the integrated medical and older people’s care sector. At this stage, the system’s primary function is that of a safety net. But, through the targeted allocation of nursing beds and healthcare and older people’s care resources, it has begun to unlock consumer demand related to health and care services.

As the pilot scheme progressed, the LTCI system gradually entered a phase of expansion and consolidation. In 2020, the National Healthcare Security Administration issued *the Guiding Opinions on Expanding the Pilot Scope of the Long-Term Care Insurance System*, extending the pilot to 29 national-level cities. This marked the system’s transition from exploratory implementation to comprehensive rollout. The scope of policy direction has expanded beyond the confines of care provision, encompassing multi-tiered models that integrate medical and older people’s care, home-based care, and digital services. This shift has not only driven functional adjustments within public healthcare institutions and older people’s care facilities, but has also accelerated the pace of private capital entering sectors such as nursing and rehabilitation. *The 14th Five-Year Plan for the Development of National Ageing Services and the Construction of the Older People’s Care Service System* proposes that by 2025, all care homes shall integrate medical and nursing services, with eligible institutions incorporated into the scope of LTCI coverage. In this process, on the one hand, LTCI directly expands the scale of health and older people’s care consumption through subsidies and service procurement. On the other hand, it has been demonstrated that the financial burden of long-term care expenses on households can be alleviated, thereby indirectly enhancing residents’ consumption capacity and willingness.

Following the 20th National Congress of the Communist Party of China, the report explicitly emphasised *“improving the Long-Term Care Insurance system”* marking LTCI as a national strategic priority and signalling a new stage of coordinated institutional development. LTCI has been advanced in conjunction with older people’s care services, public healthcare, and social capital, fostering the emergence of an integrated care system underpinned by institutional frameworks and driven by service provision. Local initiatives, including the establishment of standardised care wards, the deployment of third-party nursing services, and the implementation of Internet-enabled care models, have not only improved the quality of care services but also stimulated the growth of the nursing industry chain. Consequently, LTCI has evolved beyond a mere social security instrument to become a pivotal mechanism that promotes the interaction of institutional development, industrial growth, and consumption by optimising public resource allocation, mobilising social capital, and cultivating service demand.

### 2.2 Literature review

The World Health Organization (WHO) defines long-term care as the provision of daily assistance to individuals with limited ability for self-care, enabling them to maintain their fundamental rights, basic freedoms, and human dignity^3^. The advent of LTCI could be traced back to European countries, with the Netherlands and the United States introducing it in the 1960s and 1970s, respectively, albeit only as a component of health insurance. Since the 1990s, LTCI systems have witnessed significant advancements. Germany, through legislation, established LTCI as the sixth major insurance category, independent of health and medical insurance. Soon after this, Japan enacted *the Long-Term Care Insurance Act*, thereby incorporating LTCI into its social security system (22). Many studies focusing on countries such as the Netherlands, Japan, and South Korea have found the effects of LTCI on household assets and labour force allocation, which has triggered widespread academic interest in the socioeconomic effects of LTCI (23). Previous studies have assessed the policy effects of LTCI implementation from two main perspectives. On the one hand, from the perspective of household well-being, LTCI has been shown to alleviate both the physical and mental health burdens of care recipients and caregivers, reduce the costs of older people’s care within families, and ease financial burdens (24–28). On the other hand, from the perspective of social benefits, the implementation of LTCI helps address the issue of excessive healthcare service utilisation, enhances the efficiency of public healthcare resource allocation, and promotes the sustainability of healthcare systems. Additionally, LTCI reduces the fiscal burden on governments and improves overall social welfare (29–31). Research on LTCI in the Chinese context has primarily focused on overviews of the existing system, pointing out that China’s LTCI still faces challenges in terms of fragmented system design, fiscal sustainability, and service provision (32, 33). Meanwhile, empirical evaluation studies on pilot programs largely align with real-world experiences in other countries, indicating that LTCI significantly alleviates the burden of family caregiving, influences the use of care services, and has a notable effect on non-medical consumption among some families (34).

The existing literature that analyses the factors that influence household consumer spending considers both the individual and social environment levels. From an individual perspective, consumers’ personal characteristics and psychological factors significantly affect household consumption decisions and behaviours. Personal characteristics include gender, age, education, and income (35–38), psychological factors include self-esteem, power, stress, and self-justification strategies (39–43). From a social environment perspective, factors such as per capita GDP, total savings, inflation, public policies, and social security systems influence overall household consumption patterns (44–46).

Several studies have explored the effect of LTCI on household consumption expenditure, but have produced varying conclusions. A portion of the research argues that the LTCI system significantly incentivises household consumption expenditure. Firstly, LTCI reduces actual care-related expenses for households through subsidies. This not only fulfils the care requirements of disabled individuals and improves their health, but also lowers household medical expenses. In addition, the compensation effect of LTCI has been shown to stimulate other types of household consumption, thereby increasing overall household consumption levels (29, 34, 47). Secondly, older people are more likely to become disabled than other family members. Chinese families tend to adhere to traditional filial piety, with children assuming the obligation to support older family members, and the potential cost of their incapacitation influence the overall consumption expenditure of the family. The LTCI system helps lower older people’s care costs, alleviate fears of incapacity, reduce financial risks, and decrease the need for precautionary savings, thereby increasing household consumption (27, 48). Thirdly, home-based LTCI can enable more family members, particularly women, to be relieved from the burden of informal care-giving. This reduction in economic and psychological pressure allows them to re-enter the labour market with greater efficiency, thereby increasing household income sources and ultimately enhancing overall household consumption (49, 50). In contrast, some study contends that the LTCI system may reduce total household expenditure by diminishing overall household income. Specifically, it will lower household disposable income and precautionary savings, constrain the scale of household investment, and reduce asset returns, all of which negatively impact total household income (51–53).

Furthermore, some literature not only focuses on the direct impact of policies on insured individuals and households, but also begins to explore the spillover effects of policies transmitted through individual kinship or spatial proximity. One type of research emphasises the “nearby” spillover of LTCI on household well-being and caregiver health, indicating that LTCI can significantly improve the health and psychological conditions of the care recipients and their spouses or primary caregivers (54, 55), thereby positively affecting the labour supply and quality of life of family members (56). Another type of research focuses on the economic impact of LTCI on “local” spaces, revealing the regional aggregation of household LTCI expenditure and its high correlation with territorial policies (57). Despite these studies gradually revealing the “nearby” and “local” impacts, the existing literature still has two shortcomings. Firstly, most empirical studies focus on health, medical expenditure, or welfare indicators at the household level, lacking systematic quantification of total consumption at the city level and its effects transmitted through spatial networks. Secondly, from a macro perspective, most existing research focuses on the “local” aggregation phenomenon of LTCI’s economic effects, and discussions on “cross-local” spillover effects are still insufficient.

In summary, perspectives vary regarding how LTCI influences household consumption expenditure. However, research exploring the impact of China’s LTCI pilot program on urban residents’ consumption expenditure is still scarce, and there is a gap in discussions regarding the spatial spillover effects of LTCI. Therefore, this study examines the impact of LTCI on urban residents’ consumption and how LTCI affects urban residents’ consumption expenditure through three mechanisms—labour productivity, public service provision, and robot application. By combining a spatial econometric model, it precisely decomposes the local effects and adjacent spillovers of LTCI, enriching existing research. Utilising data from the 2011–2018 *China Migrants Dynamic Survey* (CMDS) and a DID model, this research yields several findings: Firstly, the implementation of China’s LTCI system significantly increases urban household consumption expenditure. Specifically, LTCI leads to a 7.1% rise in urban household consumer spending. Secondly, heterogeneity analysis reveals that the positive effect of LTCI on household consumption expenditure is more pronounced in cities that are net population inflow regions, where LTCI implementation results in a 7.9% increase in urban household consumption expenditure, compared to 4.4% in net population outflow regions. Furthermore, cities with higher LTCI coverage exhibit stronger effects; fully covered cities see a 95% increase in household consumption expenditure, while partially covered cities experience a 57.3% increase. Thirdly, LTCI influences urban household consumption levels through improvements in labour productivity, an increase in public healthcare service provision, and greater adoption of robotics. Extended analysis suggests that LTCI generates a spatial spillover effect, leading to higher household consumption levels in neighbouring non-pilot cities.

## 3 Theoretical analysis and hypothesis

The role of social security systems extends far beyond mere relief. In the domain of public economics, social insurance systems are recognised as fulfilling three fundamental functions. Firstly, they mitigate market failures such as information asymmetry, adverse selection, and risk-sharing issues (58). Secondly, risk-sharing is facilitated, thereby reducing household uncertainty and consequently improving consumption patterns and economic performance (59). Thirdly, they optimise resource allocation by channelling labour and capital towards more efficient destinations through institutional arrangements (60). As a vital component of social insurance, LTCI has effectively addressed the issues of information asymmetry and adverse selection within the care market through institutional arrangements. This has increased the supply of social healthcare services, alleviating the care burden on urban households, reducing income inequality and stimulating domestic consumption (51). Furthermore, the risk-sharing mechanism of LTCI reduces household care burdens and uncertainties regarding future expenditure, while its resource allocation function optimises institutional safeguards and creates additional employment opportunities. This has shaped the accumulation of health-related human capital and altered the labour supply of urban households, thereby influencing the quality of economic development and the level of urban consumption expenditure. We therefore argue that the institutional functions of the LCTI pilot scheme, as a vital component of social insurance, can stimulate urban residents’ consumption expenditure through three specific mechanisms.

Firstly, the LTCI pilot scheme enhances the supply of social healthcare services by mitigating market failures, thereby boosting consumption expenditure among urban residents. Within the care market, families encounter difficulties in accessing suitable care due to information asymmetries regarding service quality and high costs (61). The LTCI scheme has been instrumental in driving the transformation and upgrading of medical institutions, the development of integrated medical and care facilities, the expansion of nursing bed capacity, and the training of professional nursing personnel. Consequently, this has resulted in an increased provision of public services. This improves the health status of labourers, reduces health risks and uncertainties, and consequently, promotes the accumulation of health-related human capital at the urban level. According to Grossman's (62) health capital model, such accumulation contributes to extended longevity, higher labour productivity, and the promotion of high-quality economic development, ultimately resulting in increased household consumption expenditure. China’s LTCI system actively promotes the transformation and upgrading of public medical institutions, adopting an integrated medical and older people’s care model to expand the supply of older people’s care services^4^. This fully leverages the social security system’s function of mitigating market failures, directly enhancing social healthcare provision through the “visible hand.” On the one hand, the institutional development of LTCI can consolidate existing resources to optimise the allocation of healthcare resources (63). On the other hand, this policy arrangement encourages local governments to expand and establish new medical facilities, gradually increasing the number of healthcare institutions providing long-term care services, expanding relevant bed capacity, and strengthening the training and development of care personnel. Collectively, these measures enhance the overall capacity of social healthcare services. As the supply of social healthcare services expands, the stock of urban health human capital continues to grow, thereby enhancing economic quality and ultimately stimulating household consumption expenditure.

Secondly, the LTCI pilot scheme strengthens cities’ innovation capacity by risk-sharing mechanisms, thereby boosting consumption expenditure among urban residents. The boosting to innovation capacity can support high-quality economic development and stimulates additional consumer demand (64). Institutionalised pension and healthcare security schemes mitigate risks through risk-sharing mechanisms, reducing households’ exposure to health risks and uncertainty over care costs. This enables family members to participate more freely in the labour market while attracting highly skilled talent to cities with robust social security systems. By providing labour with more comprehensive pension and healthcare provisions, LTCI establishes a systemic advantage that attracts highly skilled labour from other regions (65). The inflow of these talents fosters agglomeration in cities with stronger social security conditions, thereby promoting knowledge spillovers, technological diffusion, and ultimately reinforcing urban innovation capacity. Moreover, by partially substituting for informal family care-giving, LTCI substantially alleviates household care-giving burdens, enabling more family members to participate in the labour market (66). This not only expands the overall labour supply but also increases the participation rate of skilled workers, enriching the urban talent pool and further enhancing innovation capacity. The resulting expansion of urban innovation capacity, in turn, improves the quality and resilience of economic development, ultimately leading to higher levels of household consumption expenditure.

Thirdly, the LTCI pilot scheme improves the wage levels by optimising resource allocation, thereby boosting consumption expenditure among urban residents. The development of LTCI contributes to the expansion of the older people’s care services market, creating numerous employment opportunities and increasing the overall scale of the job market (67). Moreover, LTCI releases latent demand for older adult care services among households in pilot cities by directing low-skilled workers into the care-giving industry to meet the older adult care needs of high-skilled groups, thereby facilitating more efficient matching between low- and high-skilled labour (68). Furthermore, as a government-led programme, LTCI provides low-skilled workers employed in the older adult care sector with access to employment-based health insurance and labour rights protection. This institutional design removes barriers associated with a fragmented basic medical insurance system and weak labour rights safeguards, thereby conferring institutional effectiveness upon older adult care workers seeking employment. Consequently, the labour-matching and complementary effects of LTCI are fully realised, improving the quality of high-low skill matching, contributing to higher urban wage levels, fostering high-quality economic development, and ultimately raising household consumption expenditure across cities.

**Figure 1:**
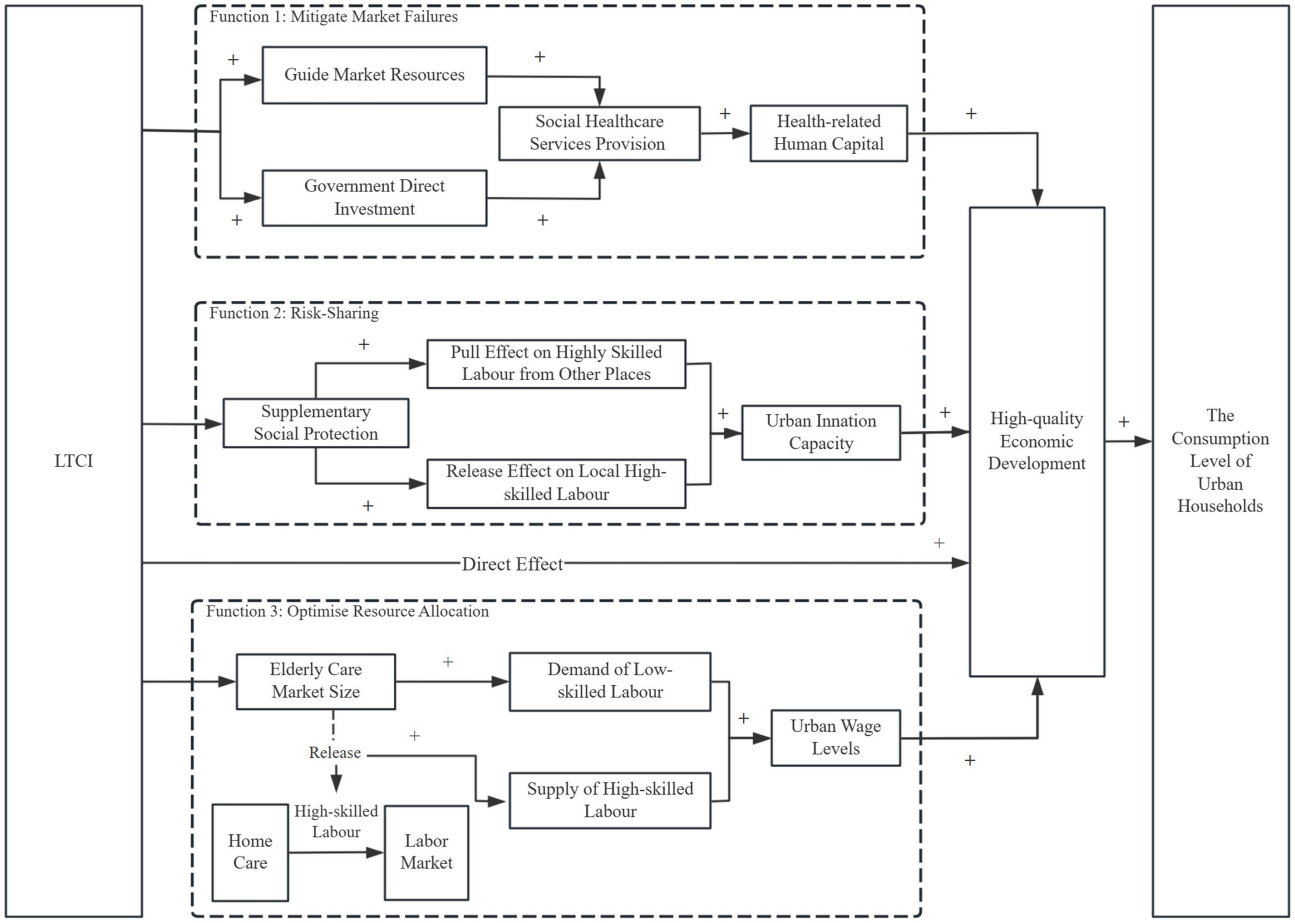
Mechanisms of LTCI impacts on urban household consumption expenditure.

Therefore, we propose the following hypotheses. The specific pathway mechanisms are illustrated in Figure 1.

*H1:* The LTCI increases the consumption level of urban households.

*H1a:* The LTCI promotes the expansion of urban household consumption expenditure by increasing the supply of social healthcare services, strengthening urban innovation capacity, and raising citywide wage levels.

The implementation of the LTCI system in China can enhance urban household consumption by boosting labour productivity, expanding public healthcare services, and promoting the use of robotics. However, the extent of these effects and their overall contribution to consumption growth are shaped by external factors such as net population migration and LTCI coverage rates, resulting in regional disparities. Firstly, in areas characterised by net population inflow, the population structure is more balanced, the productivity levels are higher, and the public facilities are more developed, which makes it more likely to gain financial support (69). These inherent advantages create better conditions for the implementation of the LTCI system, thereby amplifying the effects of labour productivity, public healthcare service provision, and the application of robotics in pilot cities, making the pilot effects more pronounced. Additionally, as urbanisation progresses, an escalating number of older individuals confronted with elevated disability risks are migrating to cities to reside with their offspring, underscoring the imperative for the LTCI system (70). Therefore, in contrast to regions experiencing net population outflow, the implementation of LTCI in areas with net population inflow is more conducive to augmenting urban household consumption levels. Secondly, the efficacy of a social security system is contingent upon the extent of its coverage, with greater coverage resulting in greater benefits for the policy’s beneficiaries and more effective implementation (71). The LTCI system boasts a comprehensive coverage base, thereby enabling its protective functions to be fully realised. This has a positive impact on urban households by increasing access to caregiving services and subsidies, thus enhancing household welfare. Therefore, the higher the coverage rate of LTCI and the larger the insured population, the more the implementation of the system helps alleviate caregiving pressures on urban households, driving urban household consumption growth. Accordingly, the subsequent hypotheses are put forward:

*H2:* In areas characterised by net population inflow, the impact of the LTCI system on urban household consumption expenditure is more pronounced.

*H3:* In pilot cities with higher coverage rates, the LTCI system exerts a more significant effect on driving urban household consumption expenditure.

## 4 Materials and methods

### 4.1 Baseline regression model

Following Wang et al. (72), we employ the DID model. The theoretical foundation of the DID model stems from the counterfactual inference framework (73), which identifies the net effect of a policy by comparing the differences in changes between the policy implementation group and the non-implementation group before and after the policy. The pilot program for LTCI has been implemented in some cities since 2016, while other regions have not yet been included in the pilot scope, forming a natural experimental group-control group structure. This grouping characteristic possesses strong exogenous characteristics, providing conditions for causal identification. From a theoretical perspective, by comparing the differences in changes between the experimental group and the control group before and after the implementation of the policy, the DID model can eliminate the interference factors of common trends on the basis of controlling for individual fixed effects and time fixed effects, thereby effectively identifying the average treatment effect of the policy. In this study, the implementation of the LTCI pilot is not random, but due to its strong exogenous characteristics, mainly stemming from the top-down selection of pilot cities by the central government, it can be reasonably regarded as an exogenous shock. Therefore, the DID method not only fits the policy background and data structural characteristics of this study but also better reflects the causal relationship between changes in consumption behaviour across different cities before and after the implementation of the policy, providing a solid econometric foundation for evaluating the economic impact of the LTCI system. This study constructs a baseline regression model using the DID method as Equation 1:

$$\mathrm{lnU}C_{\mathrm{it}}=a_{0}+\beta_{0}LT_{\mathrm{it}}+\delta_{0}X_{\mathrm{it}}+\lambda_{t}+\mu_{i}+\varepsilon_{\mathrm{it}}$$ (1)

where *i* and *t* represent cities and years, respectively; *lnUC_it_* is the dependent variable, representing the household consumption expenditure in city *i* in year *t*; *LT_it_* is the key explanatory variable, indicating the implementation of the LTCI policy. It is set to 1 for the 13 pilot cities from 2016 onwards, and 0 for the remaining 219 non-pilot cities^5^; *X_it_* represents the control variables, which include demographic, economic, and environmental factors; *t* and *i* denote year and city fixed effects, respectively; *ε_it_* represents the random error term. Taking into account potential confounding factors, this study selects control variables in terms of economic development level, regional population level, wage income, and environmental quality to reduce omitted variable bias. Furthermore, to exclude the impact of events such as social security system reforms and economic cycle fluctuations during the same period, we further introduce city fixed effects and year fixed effects for control, and policy exogenousness tests, propensity score matching difference-in-differences, and placebo tests are conducted to ensure the robustness of the estimation results and the credibility of causal interpretation in the robustness analysis.

### 4.2 Variable setting and data source

#### 4.2.1 Measure of *lnUC*

In order to ascertain the level of urban household consumption, this study selects the question from the CMDS survey: “What was your household’s average monthly total expenditure in the past year?” Following Acharya et al. (74), the average monthly total expenditure of urban households is averaged at the city level and then log-transformed.

#### 4.2.2 Measure of *LT*

**Figure 2:**
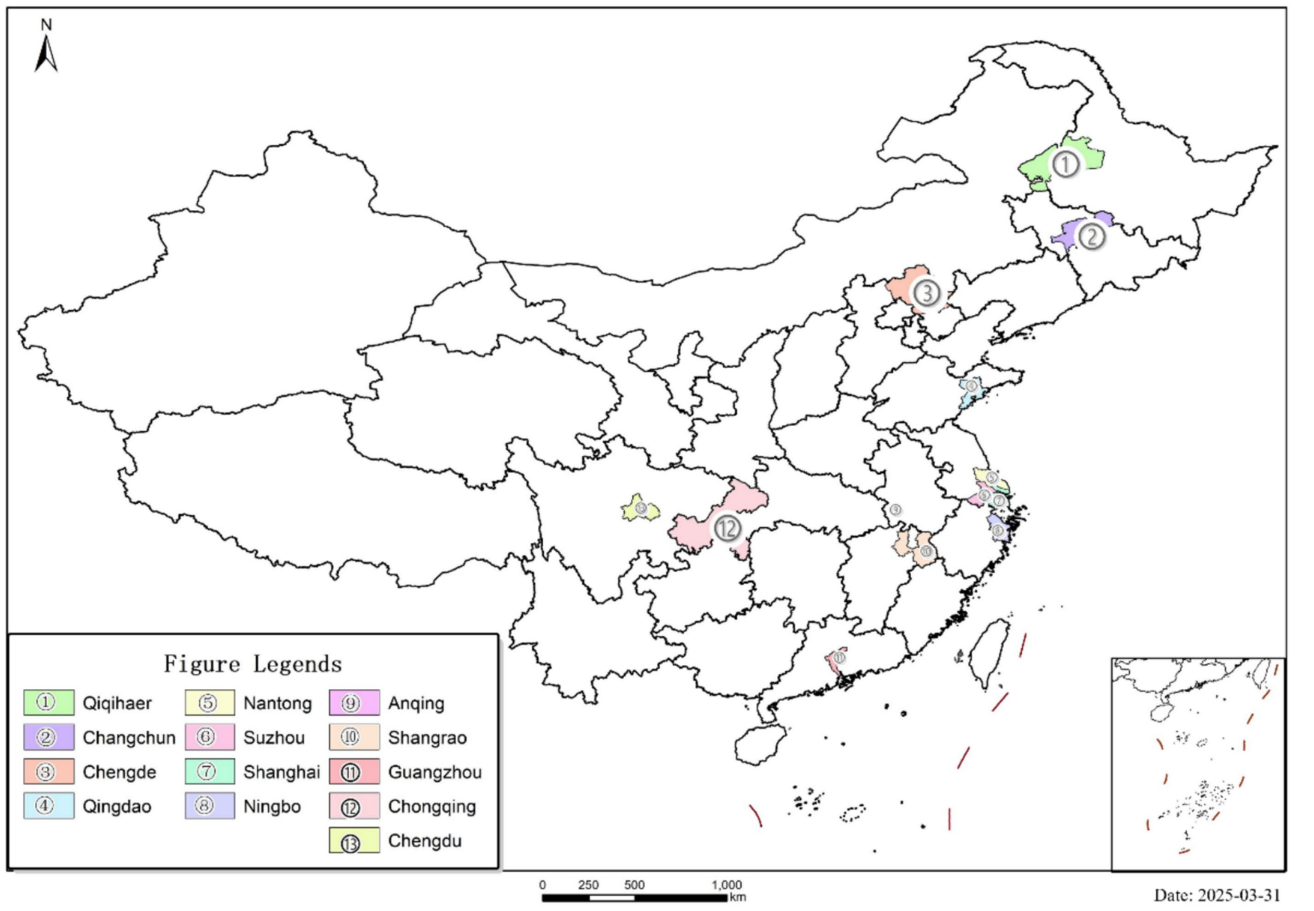
The geographical location of the specific pilot areas.

Based on the list of the first batch of national-level LTCI pilot cities in 2016, we set a dummy variable *Treat* for whether LTCI is implemented, assigning a value of 1 to the 13 pilot cities, and a value of 0 to the remaining 219 non-pilot cities. The geographical location of the specific pilot areas is illustrated in Figure 2. A policy shock time variable *Post* is set according to the pilot implementation time, where samples from 2016 onwards are assigned a value of 1, and those before are assigned a value of 0. The dependent variable in this study *LT* is the product of *Treat* and *Post*.

#### 4.2.3 Control variables

To mitigate endogeneity stemming from missing data, we control for various factors influencing urban household consumption expenditure. These factors include salary level (*Salary*), measured by the logarithm of the average wage of employees in each prefecture-level city; Tax (*Tax*), measured by the logarithm of the tax revenue level of each prefecture-level city; GDP growth rate (*GDP_growth*), represented by the growth rate of GDP; Number of people with higher education (*Edu*), represented by the logarithm of the number of household members with a high school education or above; The proportion of employees in the secondary industry (*Sec_ind_rate*), expressed as the ratio of employees in the secondary industry to the total number of employees in the primary, secondary, and tertiary industries; Population size (*Pop*), represented by the logarithm of the population density of each prefecture-level city; Number of employees (*Employees*), expressed by the logarithm of the average number of all on-the-job employees in each prefecture-level city; Urban pollution level (*Pollution*), calculated using the entropy method, incorporating emissions from industrial sulphur dioxide, solid waste, and domestic sewage.

After implementing these adjustments, we utilise panel data from 232 Chinese cities spanning 2011 to 2018 as our research sample. Given data constraints and the need for sample comparability, we designate 13 cities as LTCI pilot sites. The data primarily come from the CMDS and the China City Statistical Yearbook. The CMDS has been collected by the National Health Commission of China since 2009. This survey employs a multi-stage, multi-level probability sampling method proportional to the labour force size. It covers 319 cities across China, excluding Hong Kong, Macao, and Taiwan, as well as the Xinjiang Production and Construction Corps. The dataset encompasses information on the basic characteristics of urban households, employment, housing, health, and social integration, providing essential data for this study. Table 1 provides descriptive statistics for the variables. As the focal point of this study is urban households, all CMDS variables are aggregated to the city level. The remaining necessary city characteristic variables are sourced from the China City Statistical Yearbook and are matched to the CMDS data by city. In order to ensure that the sample information fits the defined scope of this study, the 2011–2018 A-series CMDS survey data was selected as the research sample, with missing key variable data being excluded. Data from 232 cities are retained, resulting in a final panel dataset of 1,856 total samples. This comprehensive dataset provides a reliable and accurate reflection of economic development, demographic characteristics and environmental quality of these cities, thereby ensuring the validity and reliability of our results.

**Table 1:** Summary descriptive statistics.

Variable	*N*	Mean	Std. dev.	Min	Max
Dependent variables					
*lnUC*	1856	7.894	0.296	6.544	10.330
Independent variables					
*LT*	1856	0.021	0.143	0.000	1.000
Mechanism variables					
*H_wage*	1854	2.997	0.296	1.988	8.966
*M_income*	1854	8.103	0.310	7.001	14.125
*Doctors*	1856	1.075	1.052	0.078	10.938
*Beds*	1856	2.122	1.903	0.135	17.741
*R_stock*	1832	16.844	16.962	0.229	108.063
*R_install*	1832	4.786	4.366	0.101	25.911
Control variables					
*Salary*	1856	10.850	0.295	8.509	11.920
*Tax*	1856	8.170	0.890	5.791	11.310
*GDP_growth*	1856	8.981	4.280	−15.950	109.000
*Edu*	1856	4.606	0.573	0.588	6.386
*Sec_ind_rate*	1856	0.454	0.145	0.045	0.844
*Pop*	1852	5.891	0.710	3.054	8.131
*Employees*	1856	3.744	0.859	1.810	6.896
*Pollution*	1856	0.079	0.082	0.000	0.732

## 5 Empirical results

### 5.1 Baseline regression analysis

The baseline regression results are presented in Table 2, where columns (1) to (3) display the results with the gradual inclusion of control variables and both time and city fixed effects. It can be observed that after including the control variables, the results remain statistically significant at the 1% level. After controlling for both time and city fixed effects, the results remain significant at the 5% level. This finding lends support to Hypothesis 1, which posits that, in comparison to cities devoid of LTCI pilots, cities with the pilot have exhibited a substantial augmentation in urban household consumption levels. Subsequent analysis will delve into the specific mechanisms that underpin this phenomenon. To circumvent the potential overestimation of the impact of the long-term care insurance pilot on urban household consumption, columns (4) to (6) incorporate three policy dummy variables: Health City, National Information Consumption City, and Carbon Emissions Trading City. In column (7), all three of these policy dummy variables are included simultaneously to exclude potential confounding effects from other policies. The results demonstrate that the LTCI pilot continues to significantly enhance urban household consumption levels.

**Table 2:** Baseline regression results.

Variables	Dependent variable: urban household consumption (*lnUC*)						
	(1)	(2)	(3)	(4)	(5)	(6)	(7)
*LT*	0.313*** (6.60)	0.086** (2.24)	0.071** (2.51)	0.066** (2.28)	0.071** (2.49)	0.071** (2.49)	0.065** (2.24)
*Policy_HC*				0.018 (0.92)			0.020 (1.03)
*Policy_ICC*					−0.146*** (−4.31)		−0.150*** (−4.76)
*Policy_CERP*						−0.007 (−0.18)	−0.009 (−0.49)
*Salary*		0.594*** (24.29)	0.121** (2.15)	0.122** (2.16)	0.121** (2.15)	0.122** (2.16)	0.122** (2.17)
*Tax*		0.068*** (5.24)	0.092*** (2.88)	0.090*** (2.86)	0.094*** (2.95)	0.091*** (2.88)	0.093*** (2.92)
*GDP_growth*		−0.005*** (−3.94)	−0.003** (−2.34)	−0.003** (−2.35)	−0.003** (−2.34)	−0.003** (−2.38)	−0.003** (−2.38)
*Edu*		−0.013 (−1.27)	0.027* (1.82)	0.027* (1.75)	0.027* (1.80)	0.027* (1.82)	0.026* (1.73)
*Sec_ind_rate*		−0.060 (−1.45)	−0.127 (−1.16)	−0.122 (−1.13)	−0.126 (−1.16)	−0.126 (−1.16)	−0.122 (−1.13)
*Pop*		0.106*** (6.35)	0.179* (1.70)	0.177* (1.69)	0.174* (1.67)	0.180* (1.70)	0.174* (1.67)
*Employees*		−0.097*** (−5.95)	0.045* (1.77)	0.046* (1.80)	0.044* (1.72)	0.046* (1.77)	0.046* (1.77)
*Pollution*		−0.159** (−2.17)	−0.037 (−0.57)	−0.044 (−0.66)	−0.041 (−0.63)	−0.036 (−0.56)	−0.049 (−0.73)
*Constant*	7.888*** (1,148.28)	0.771*** (3.03)	4.309*** (5.15)	4.313*** (5.15)	4.384*** (5.27)	4.296*** (5.10)	4.372*** (5.21)
*City FE*	No	No	Yes	Yes	Yes	Yes	Yes
*Year FE*	No	No	Yes	Yes	Yes	Yes	Yes
*N*	1,856	1,852	1,852	1,852	1,852	1,852	1,852
*R^2^*	0.023	0.439	0.679	0.679	0.680	0.679	0.680

*** *p* < 0.01, ** *p* < 0.05, and * *p* < 0.10. T values are in parentheses.

### 5.2 Robustness check

#### 5.2.1 Parallel trend test

The parallel trend assumption is essential for applying DID. Before implementing the LTCI system, it is essential that both the control group cities (without the pilot) and the treatment group cities (with the pilot) show the same trend in urban household consumption expenditure. Because the parallel trend assumption holds, the observed variations in the dependent variable can be causally attributed to the LTCI policy intervention. In order to ascertain whether the treatment and control groups exhibited parallel trends in household consumption expenditure prior to the implementation of the LTCI pilot policy, we employ an event study approach (ESA). Following the methods outlined by Qi et al. (75) and Wang et al. (76), the year prior to the policy shock, 2015, is designated as the baseline for the parallel trend test. The Equation 2 is established:

$$\mathrm{lnU}C_{\mathrm{it}}=\alpha +\sum_{k=-5,i\neq -1}^{2}\beta_{k}LT_{i,t+k}+\lambda X_{\mathrm{it}}+\eta_{t}+\upsilon_{i}+\varepsilon_{\mathrm{it}}$$ (2)

where *LT_k_* represents a series of dummy variables, and *k* denotes the number of years since the implementation of the LTCI pilot policy. Other variables remain the same as in Equation 1. Specifically, *k* equals zero represents the year in which the LTCI pilot was implemented, *k* equals one indicates the first year after implementation of LTCI, and k equals negative one refers to the year before implementation of LTCI, and so forth. The coefficient *β_k_* is the primary focus of this study, as it measures the impact of LTCI pilot implementation on urban household consumption levels in year *k*.

**Figure 3:**
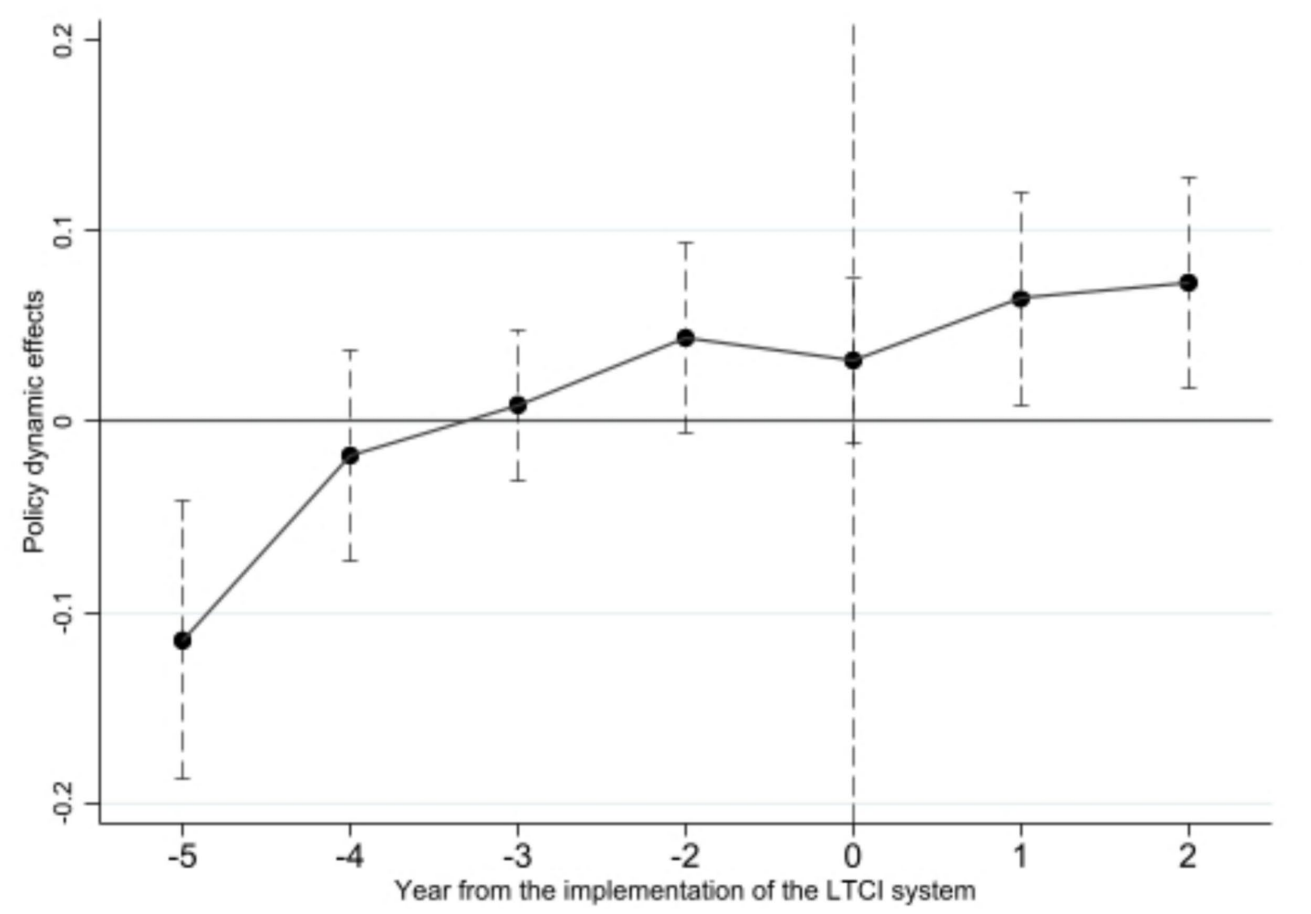
Parallel trend test.

As demonstrated in Figure 3, the outcomes of the parallel trend test reveal that prior to the implementation of the LTCI system, the estimated coefficients of urban household consumption expenditure for both the treatment and control groups exhibited fluctuations around zero, with a consistent trend. In the second year following the policy implementation, the estimated results for the treatment and control groups significantly deviated from zero and demonstrated an upward trend. This finding suggests that following the implementation of the LTCI system, a marked divergence emerged in the consumption expenditure trends of the two groups, although the policy effect demonstrated a certain time lag. The parallel trend assumption is thus validated. It is noteworthy that the event-study results reveal a significantly negative coefficient for 2011. We argue that this anomaly should not be interpreted as evidence of an adverse effect of the LTCI pilot on household consumption, but rather as a consequence of price fluctuations in the economy during that year. According to data released by the National Bureau of Statistics, China’s CPI in 2011 recorded a year-on-year increase that was markedly higher than in the preceding and subsequent years (3.3% in 2010, 5.4% in 2011, and 2.6% in 2012), with the primary driving factor being the abnormal surge in food prices. From the supply-side perspective, this surge was underpinned by a temporary shortage in food supply, most notably reflected in the sharp reduction of hog slaughter in certain regions, with comparable patterns observed across other cities. This shortage precipitated a pronounced rise in food prices, which was subsequently transmitted to the overall level of consumer prices. Prior research suggests that inflation volatility and uncertainty exert a significant restraining effect on household consumption, prompting families to adopt greater caution in their expenditure patterns (77). To lend further support to this interpretation, we compiled CPI and food price index data for the treatment cities over the period 2010–2012 and computed the variance as a measure of price volatility. The findings reveal that price fluctuations in the treatment group averaged 220.17%, exceeding the national average of 212.33%, thereby indicating the presence of asymmetric price shocks between the treatment and control groups in the pre-treatment period. This evidence suggests that the anomalous negative coefficient observed in 2011 reflects pre-existing macroeconomic shocks rather than a negative effect of the long-term care insurance pilot on household consumption. This asymmetry may have led to systematic differences in the consumption behaviour of residents in the treatment group relative to the control group in 2011, thereby manifesting as an outlier in a single year within the parallel trend test. However, this does not undermine the validity of the overall parallel trend assumption, nor does it compromise our empirical conclusion regarding the positive effect of the long-term care insurance policy.

#### 5.2.2 Policy exogeneity tests

In the selection of pilot cities for the LTCI scheme, policymakers may favour cities with higher pre-existing consumption levels, thereby ensuring optimal acceptance and utilisation of LTCI polit policy. To assess whether the policy satisfies the exogeneity assumption, we examine the correlation between the treatment variable (*Treat*) and urban household consumption levels. Following the approach of Qin et al. (78), which utilises five pre-policy implementation sample periods (2015, 2014, 2013, 2012, and 2011) to establish the Equation 3:

$$\text{Trea}t_{\mathrm{it}}=\delta_{0}+\text{ρlnU}C_{i}+\lambda \sum X_{i}+\varepsilon_{\mathrm{it}}$$ (3)

where *Treat_i_* represents the grouping variable for the treatment and control groups, and *ρ* is the variable coefficient. After pre-processing the data, we retain five years of pre-policy cross-sectional observations (2011–2015) with no temporal variation. To capture the treatment effect, dummy variables (1 for treated cities, 0 otherwise) are constructed. We then estimate the Probit model to assess the policy impact. The policy exogeneity test results are shown in Table 3, where the five-period regression outcomes for *lnUC* are statistically insignificant. This indicates that urban household consumption levels in pilot cities do not influence the LTCI pilot’s implementation, thus confirming the policy exogeneity test was successfully passed.

**Table 3:** Policy exogeneity test.

Variables	Dependent variable: grouping variables for treatment and control groups (*treat*)				
	Year: 2015	Year: 2014	Year: 2013	Year: 2012	Year: 2011
	(1)	(2)	(3)	(4)	(5)
*lnUC*	0.053 (0.11)	1.675 (1.58)	0.718 (0.88)	0.078 (0.11)	−0.916 (−1.33)
*Salary*	−0.184 (−0.37)	0.541 (0.41)	2.124 (1.60)	0.052 (0.03)	0.221 (0.16)
*Tax*	0.232 (0.62)	0.195 (0.38)	−0.285 (−0.57)	1.033** (2.09)	1.222** (2.43)
*GDP_growth*	−0.047 (−1.25)	−0.124*** (−2.65)	−0.049 (−1.00)	−0.100 (−1.39)	−0.023 (−0.29)
*Edu*	−0.227 (−0.66)	−0.337 (−1.01)	−0.592* (−1.65)	−0.802* (−1.81)	−0.572 (−1.49)
*Sec_ind_rate*	−0.696 (−0.44)	0.702 (0.35)	0.182 (0.10)	0.097 (0.05)	−0.344 (−0.17)
*Pop*	1.025 (1.62)	1.577** (2.30)	0.914 (1.40)	2.519*** (3.14)	2.477*** (2.82)
*Employees*	0.313 (0.59)	0.003 (0.01)	0.417 (0.71)	−0.828 (−1.42)	−0.911 (−1.39)
*Pollution*	0.534 (0.27)	−0.424 (−0.25)	0.504 (0.21)	−1.251 (−0.42)	−2.326 (−0.64)
*Constant*	−8.048 (−0.97)	−29.988* (−1.71)	−32.120** (−1.98)	−18.684 (−1.27)	−15.369 (−1.07)
*City FE*	-	-	-	-	-
*Year FE*	-	-	-	-	-
*N*	232	232	232	232	231
*Pseudo R^2^*	0.306	0.355	0.351	0.379	0.406

*** *p* < 0.01, ** *p* < 0.05, and * *p* < 0.10. T values are in parentheses. Z-statistics are in parentheses, and the corresponding R^2^ is pseudo R^2^.

#### 5.2.3 Replacing the dependent variable

Following the methodology of Waehrer (79) for analysing non-health-related consumption, we employ food expenditure and housing expenditure as proxies. The approach involves re-testing the variables by sequentially substituting urban household consumption expenditure with urban household food expenditure and then with urban household housing expenditure. The regression results in columns (1) and (2) of Table 4 indicate that pilot cities exhibit significantly higher food and housing expenditures than non-pilot cities, confirming the baseline findings and supporting the reliability of this study.

**Table 4:** Robustness test.

Variables	Dependent variable				
	*lnSPZC*	*lnZFZC*	*lnUC*		
	Replace the explained variable		Replace sample		PSM-DID
	(1)	(2)	(3)	(4)	(5)
*LT*	0.066** (2.36)	0.184*** (2.45)	0.062** (2.50)	0.073** (2.39)	0.055* (1.66)
*Salary*	0.096* (1.93)	0.154* (1.01)	0.148** (2.06)	0.121** (2.15)	0.123** (2.14)
*Tax*	0.059* (1.78)	0.369*** (5.16)	0.082** (2.43)	0.092*** (2.89)	0.088*** (2.63)
*GDP_growth*	−0.001 (−0.98)	−0.007** (−2.31)	−0.002 (−0.74)	−0.003** (−2.36)	−0.003** (−2.14)
*Edu*	0.057*** (3.62)	0.013 (0.24)	0.036** (2.06)	0.027* (1.81)	0.030 (1.49)
*Sec_ind_rate*	0.092 (0.86)	−0.398** (−1.80)	−0.144 (−1.49)	−0.125 (−1.15)	−0.206* (−0.80)
*Pop*	0.104 (0.99)	0.686*** (3.42)	0.124 (1.37)	0.179* (1.70)	0.197 (1.41)
*Employees*	0.008 (0.38)	0.219*** (2.81)	0.047* (1.90)	0.045* (1.77)	0.056** (2.02)
*Pollution*	−0.166** (−2.51)	0.121 (0.75)	−0.046 (−0.59)	−0.038 (−0.58)	−0.048 (−0.67)
*Constant*	4.396*** (6.07)	−2.219 (−0.96)	4.376*** (5.06)	4.309*** (5.15)	4.180*** (3.98)
*City FE*	Yes	Yes	Yes	Yes	Yes
*Year FE*	Yes	Yes	Yes	Yes	Yes
*Province FE*					
*N*	1,850	1,850	1,852	1,852	1,685
*R^2^*	0.924	0.652	0.721	0.679	0.676

*** *p* < 0.01, ** *p* < 0.05, and * *p* < 0.10. T values are in parentheses.

#### 5.2.4 Changing the sample

First, we winsorise the sample by trimming observations below the 1st and above the 99th percentiles. The regression results of the new sample are presented in column (3) of Table 4, indicating that the implementation of LTCI continues to significantly enhance urban household consumption expenditure. The second approach involves the exclusion of Qingdao from the sample and the re-running of the regression. In June 2016, Chinese General Office of the Ministry of Human Resources and Social Security issued *the Guiding Opinions on the Pilot Implementation of the Long-Term Care Insurance System*, officially designating Qingdao as a national pilot city. However, it should be noted that Qingdao had independently implemented the LTCI system in 2012, with notable results (80). Considering the potential impact of this particularity on the reliability of this study, we excluded Qingdao from the 13 pilot cities, constructing a new treatment group with the remaining 12 pilot cities and re-running the regression to test the robustness of our findings. The results in column (4) of Table 3 show that, even after excluding the early pilot in Qingdao, the LTCI system still significantly impacts the urban household consumption expenditure, supporting the conclusions of this study.

#### 5.2.5 Propensity score matching

To address selection bias, we employ Propensity Score Matching (PSM) combined with DID. This ensures balanced covariates between treatment and control groups prior to the LTCI pilot. Using the logit model and all control variables as predictors, the nearest-neighbour matching method with a caliper width of 0.01 is applied to select cities from the control group with propensity scores closest to those in the experimental group. Based on the matched control and experimental groups, the regression is re-run using Equation 1. The results in column (5) of Table 4 show that the LTCI system still significantly increases the consumption expenditure levels of urban households, confirming the reliability of the conclusions in this study. Additionally, to confirm the reliability of the propensity score matching test, the matching results are verified by a balance test the balance test results are shown in Appendix, where after matching, the standardised bias of most control variables is less than 5%, and the t-test results are not significant, indicating that the balance test passes and the matching is satisfactory.

#### 5.2.6 Placebo test

**Figure 4:**
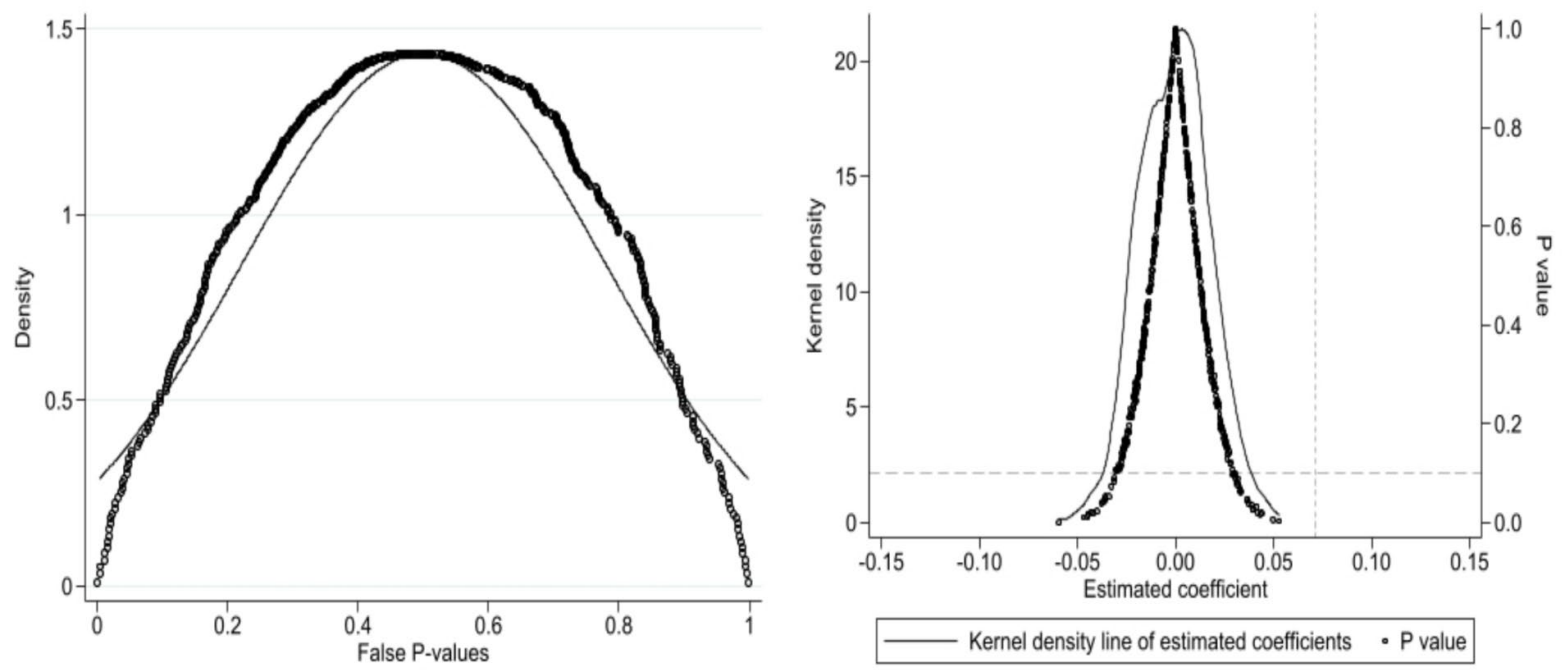
Placebo test distribution chart.

We conduct a placebo test by randomly assigning LTCI status to check if the impact of LTCI system on consumption is caused by any unobserved factors. Specifically, drawing on the approach of Liu and Lu (81), we select the same number of cities as the treatment group to form a pseudo-treatment group. With control variables and model construction methods remaining unchanged, we randomly conduct 500 parameter estimations identical to the baseline regression, and plot the distribution of false *p*-values and false estimated coefficients. The results are shown in Figure 4. The left and right figures are the distribution of false p-values and false estimated coefficients, respectively, indicating that the majority of the fictitious p-values are greater than the critical value of zero, and the fictitious estimated coefficients are approximately normally distributed, centred around zero. In contrast, the true regression coefficients significantly differ from the fictitious estimated coefficients, confirming that the effect of LTCI on household consumption is not driven by unobserved factors.

#### 5.2.7 Micro-level analysis

To further enhance the analytical granularity of this study, we re-estimate the model at the micro-individual level by reconstructing the regression framework with variables pertaining to LTCI, household consumption, and associated factors. Given that the CMDS does not constitute a continuous panel dataset, and in order to ensure methodological rigour and scientific validity, we adopt the procedure proposed by Zhang et al. (82). Specifically, a PSM technique is applied to conduct 1:1 annual matching, thereby converting the non-panel dataset into a pseudo-panel structure suitable for regression analysis. In operational terms, household consumption is peroxided by the logarithm of monthly household expenditure (*lnExpense*), whilst residents’ income is measured by the logarithm of hourly wages in the preceding month (*ln_hour*). Moreover, consistent with Hatef et al. (83), individuals’ cognitive perception of public service provision is utilised as a proxy for the actual level of public service supply. The rationale is that where public services are sufficient and of relatively high quality, residents are more likely to perceive and acknowledge their presence; conversely, inadequate or uneven provision is readily discernible in daily life and thus reflected in lower levels of awareness. Accordingly, we employ residents’ awareness of health records (*Health*) as the proxy variable: individuals who have established or are aware of such records are coded as 1, and all others are coded as 0. In addition, a comprehensive set of individual-level controls is introduced, including age (*Age*), educational attainment (*Edu*), employment status (*Emp_status*), marital status (*Marital*), nature of employment (*Nature_emp*), household size (*Family_num*), regional GDP level (*lnGDP*), and investment in science and technology (*lnF_expense_S*). The regression estimates reported in Table 5 demonstrate that, even at the micro-individual level, the pilot implementation of LTCI exerts a statistically significant and robust positive effect on household consumption. The underlying mechanisms operate primarily through the expansion of public service provision and the enhancement of residents’ income levels, thereby confirming the validity of the baseline results.

**Table 5:** Micro-level analysis.

Variables	Dependent variable		
	*lnExpense*	*Health*	*In_hour*
	(1)	(2)	(3)
*LT*	0.030** (2.42)	0.062*** (2.92)	1.345** (2.22)
*Age*	0.000 (0.79)	0.000 (0.47)	0.084*** (3.71)
*Edu*	0.127*** (41.49)	−0.034*** (−7.10)	4.283*** (24.85)
*Emp_status*	0.060*** (24.77)	−0.006 (−1.45)	0.871*** (5.69)
*Marital*	0.026*** (5.88)	0.024*** (3.24)	0.774*** (3.02)
*Nature_emp*	−0.015*** (−13.56)	0.009*** (4.42)	−0.001 (−0.02)
*Family_num*	0.177*** (68.83)	−0.005 (−1.26)	0.403** (2.47)
*lnGDP*	−0.020*** (−2.71)	0.037*** (3.09)	−2.102*** (−5.86)
*lnF_expense_S*	0.066*** (14.02)	−0.011 (−1.42)	2.743*** (10.61)
*Constant*	6.526*** (77.11)	−0.343** (−2.47)	4.721 (1.09)
*City FE*	Yes	Yes	Yes
*Year FE*	Yes	Yes	Yes
*N*	16,053	16,032	16,052
*R^2^*	0.187	0.005	0.021

*** *p* < 0.01, ** *p* < 0.05, and * *p* < 0.10. T values are in parentheses.

### 5.3 Heterogeneity test

#### 5.3.1 Regions of net population inflow and outflow

**Figure 5:**
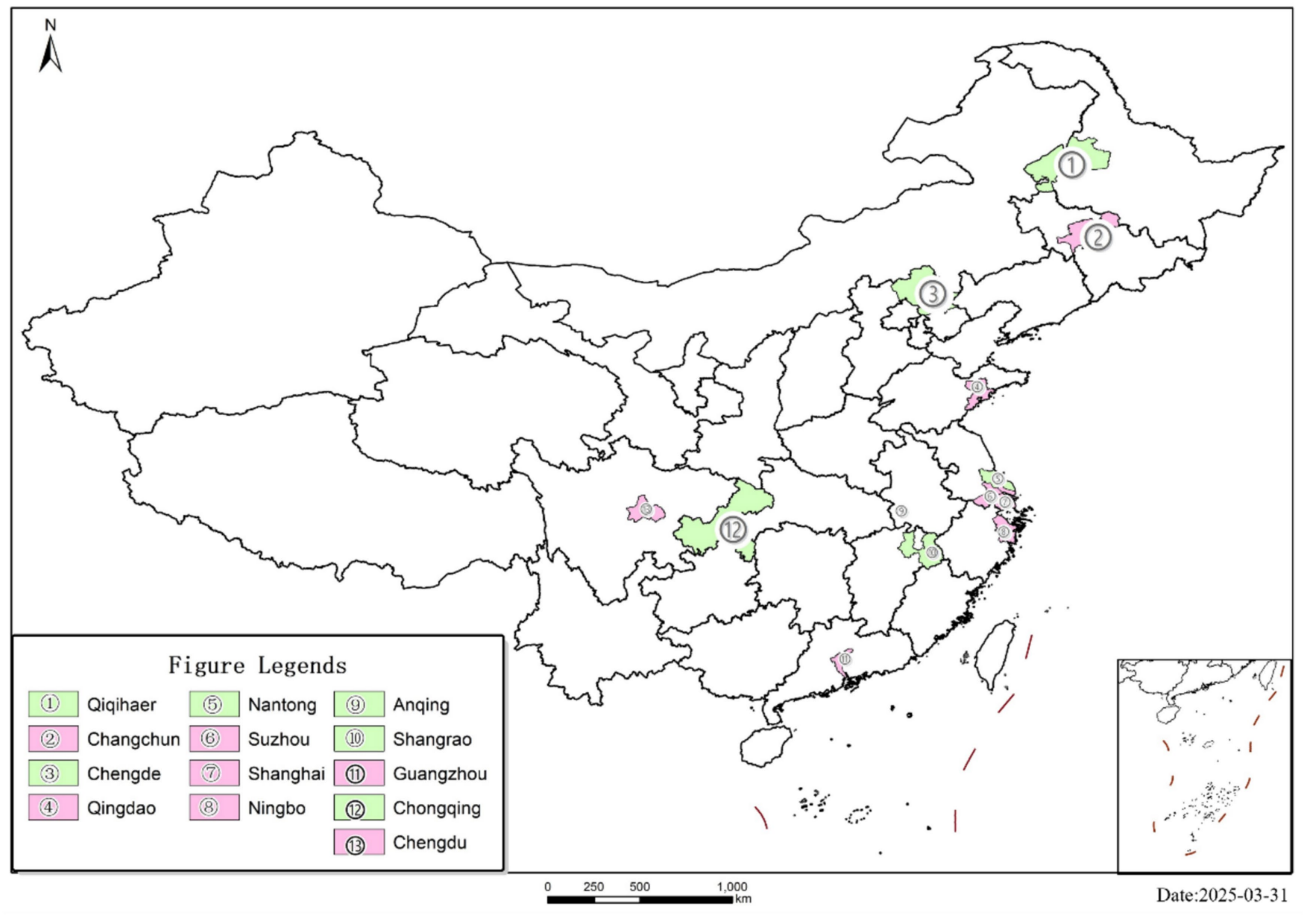
Regions of net population inflow and outflow.

The implementation of the LTCI system is more efficient in regions with a more rational demographic structure, higher levels of productivity, and superior financial resources, which are uniquely positioned to implement the policy. In net population inflow regions, the LTCI pilot leverages both favourable implementation conditions and urbanisation-induced older migration. These dynamics not only enhance policy feasibility but also elevate public awareness of the LTCI system. This increased awareness has led to greater prioritisation and support for LTCI, thereby strengthening the influence of the pilot LTCI system on urban household consumption expenditure. In contrast, factors such as limited financial resources, low productivity, and significant labour loss may reduce the impact of the LTCI pilot on urban household consumption expenditure in areas with net population outflows. To validate this view, we explore the heterogeneity of LTCI implementation based on net inflow and net outflow regions. Specially, the districts are divided as shown in Figure 5, where pink areas represent areas of net population inflow and green areas represent areas of net population outflow. The results in columns (1) and (2) of Table 5 reveal that the LTCI system significantly increases urban household consumption expenditure in regions with net inflows. In contrast, the results are not significant for net outflow regions, supporting H2.

#### 5.3.2 Coverage area

**Figure 6:**
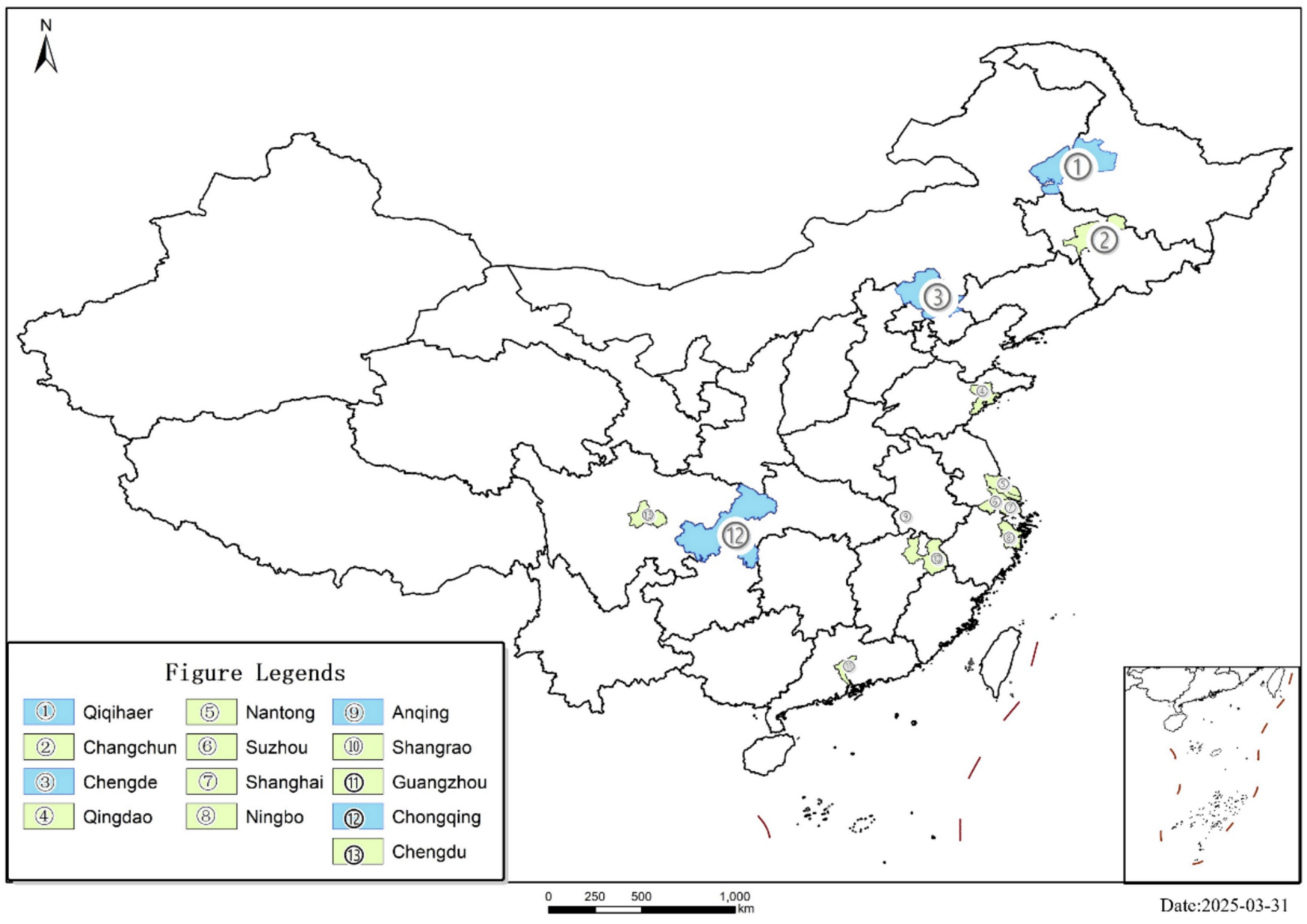
Coverage area.

In China, the two main types of basic medical insurance for urban residents are the Urban Employee Basic Medical Insurance (UEBMI) and the Urban–Rural Resident Basic Medical Insurance (URRBMI). The UEBMI covers employees in formal sectors, while the URRBMI covers other residents in both urban and rural areas (84). Given the varying coverage of LTCI in pilot cities in China, following the principle of gradual expansion, cities have chosen their coverage scope based on local characteristics. Among the 13 LTCI pilot cities launched in 2016, all except Chengde, Qiqihar, Anqing, and Chongqing extend coverage to both UEBMI and URRBMI enrolees. The four excluded cities only cover UEBMI enrolees. We designate Chengde, Qiqihar, Anqing, and Chongqing as the partial coverage group, while the remaining pilot cities are designated as the full coverage group for the purpose of heterogeneity analysis. Specially, the districts are divided as shown in Figure 6, where the green areas represent the full coverage cities and the blue areas represent the partial coverage cities. The results, as illustrated in columns (3) and (4) of Table 6, demonstrate that, in comparison to the partial coverage group, the full coverage group exhibits more substantial outcomes, thereby suggesting that pilot cities with higher LTCI coverage possess more pronounced policy implications. One potential explanation for this finding is that, in comparison to pilot cities with partial coverage, cities with full coverage have a more extensive beneficiary population. A greater proportion of urban residents’ benefit from the subsidies and services of LTCI, resulting in a more substantial increase in disposable income and a diminished preventive savings motive, which more effectively promotes an increase in urban household consumption levels.

**Table 6:** Heterogeneity analysis.

Variables	Dependent variable: urban household consumption (*lnUC*)			
	Nature of population mobility		Coverage scope	
	Net population outflow areas	Net population inflow areas	The partial coverage group	The full coverage group
	(1)	(2)	(3)	(4)
*LT*	0.044 (0.91)	0.079** (2.28)	0.048 (0.89)	0.080** (2.55)
*Salary*	0.088 (1.54)	0.235*** (2.83)	0.116** (2.05)	0.133** (2.11)
*Tax*	0.047 (1.17)	0.153*** (3.27)	0.093*** (0.92)	0.096*** (2.95)
*GDP_growth*	0.000 (0.12)	−0.004*** (−6.46)	−0.003** (−2.43)	−0.003** (−2.32)
*Edu*	0.025 (1.34)	0.005 (0.21)	0.028* (1.83)	0.023 (1.52)
*Sec_ind_rate*	−0.169 (−1.10)	−0.027 (−0.19)	−0.163 (−1.43)	−0.138 (−1.24)
*Pop*	0.408* (1.96)	0.053 (0.81)	0.187* (1.69)	0.181* (1.67)
*Employees*	0.045 (1.25)	0.038 (1.11)	0.034 (1.31)	0.048** (1.82)
*Pollution*	−0.051 (−0.48)	−0.012 (−0.14)	−0.042 (−0.61)	−0.037 (−0.56)
*Constant*	3.659*** (2.74)	3.340*** (3.44)	4.626*** (5.21)	4.404*** (4.64)
*City FE*	Yes	Yes	Yes	Yes
*Year FE*	Yes	Yes	Yes	Yes
*N*	1,143	709	1782	1820
*R^2^*	0.598	0.860	0.767	0.773

*** *p* < 0.01, ** *p* < 0.05, and * *p* < 0.10. T values are in parentheses.

### 5.4 Mechanism analysis

The previous analysis shows that the LTCI system implementation notably boosts urban household consumption levels. How does the LTCI system induce urban households to consume more? In addressing this question, drawing on the research of Chen et al. (85) and Dell (86), we employ the two-step method to examine the mechanisms through which the LTCI pilot drives urban household consumption expenditure, focusing on labour productivity, public service provision, and the extent of robot application.

#### 5.4.1 The provision of social healthcare services

The pilot of the LTCI system boosts urban household consumption levels by increasing the supply of social healthcare services. The provision of public services affects the accumulation of health-related human capital, which constitutes a key driver of high-quality economic development. Expanding the scope of public service provision promotes the accumulation of human capital, thereby enhancing the level of economic development and significantly increasing urban household consumption expenditure in pilot regions. To assess this dynamic mechanism, we use the number of doctors in urban areas (in 10,000 units) and the number of hospital and clinic beds (in 10,000 units) which are denoted as *Doctors* and *Beds* respectively, as alternative indicators for the supply of public healthcare services. These two indicators are used as dependent variables to re-conduct the DID regression. The regression results are presented in columns (1) and (2) of Table 7. The LTCI system’s implementation notably boosted public healthcare services in cities, with an increase of 5,188 doctors and 7,732 beds. This suggests that the LTCI system stimulates urban household consumption expenditure by increasing the supply of social healthcare services.

**Table 7:** Mechanism testing.

Variables	Dependent variable					
	*Doctors*	*Beds*	*R_stock*	*R_install*	*H_wage*	*M_income*
	Social healthcare services provision		urban innovation capacity		Urban wage levels	
	(1)	(2)	(3)	(4)	(5)	(6)
*LT*	0.519*** (2.96)	0.773*** (2.87)	0.077** (2.02)	0.018** (2.00)	0.073*** (2.91)	0.075*** (2.87)
*Salary*	0.014 (0.25)	0.140 (1.32)	−0.035 (−1.13)	−0.007 (−0.98)	0.090* (1.71)	0.096* (1.73)
*Tax*	0.130*** (2.80)	0.192*** (2.88)	0.027 (1.45)	0.006 (1.33)	0.135*** (4.58)	0.142*** (4.56)
*GDP_growth*	0.003 (1.16)	0.002 (0.73)	0.001 (1.21)	0.000 (1.44)	0.000 (0.18)	0.000 (0.20)
*Edu*	0.044 (1.51)	0.093** (2.26)	−0.005 (−0.41)	−0.001 (−0.36)	0.032* (1.75)	0.034* (1.71)
*Sec_ind_rate*	−0.567** (−2.59)	−0.862** (−2.22)	−0.204*** (−2.82)	−0.054*** (−2.95)	−0.054 (−0.56)	−0.052 (−0.50)
*Pop*	0.758*** (2.67)	1.125*** (2.77)	0.172** (2.19)	0.039** (2.05)	0.215*** (2.98)	0.221*** (2.96)
*Employees*	0.104** (2.01)	0.227*** (2.76)	0.054*** (2.70)	0.015*** (2.86)	0.061** (2.11)	0.064** (2.08)
*Pollution*	0.051 (0.40)	0.489 (1.48)	0.197*** (3.00)	0.059*** (3.29)	−0.056 (−0.92)	−0.062 (−0.96)
*Constant*	−5.087*** (−2.76)	−8.810*** (−3.14)	−0.908 (−1.50)	0.197 (3.00)	−1.019 (−1.40)	3.892*** (5.07)
*City FE*	Yes	Yes	Yes	Yes	Yes	Yes
*Year FE*	Yes	Yes	Yes	Yes	Yes	Yes
*N*	1,852	1,852	1832	1832	1,850	1,850
*R^2^*	0.320	0.431	0.780	0.787	0.520	0.533

*** *p* < 0.01, ** *p* < 0.05, and * *p* < 0.10. T values are in parentheses.

#### 5.4.2 The urban innovation capacity

The LTCI pilot promotes urban household consumption expenditure by enhancing the urban innovation capacity. The LTCI can increase the supply of high-skilled labour in pilot cities through two channels: by attracting highly skilled talent from other regions and by releasing the potential of local high-skilled workers. The resulting expansion of high-skilled labour contributes to the enhancement of urban innovation capacity. Improvements in urban innovation capacity, in turn, facilitate high-quality economic development and stimulate additional consumer demand. To test this mechanism, following the research design of Adler and Florida (87), we use the stock density (*R_stock*) and installation density of robots (*R_install*) in various cities as alternative indicators for the urban innovation capacity, and re-estimate the DID regression using these two indicators as dependent variables. Columns (3) and (4) of Table 7 indicate that the LTCI system’s implementation significantly enhanced robot adoption, raising robot stock density by 7.7% and installation density by 1.8%. This indicates that the LTCI system promotes urban household consumption growth by enhancing the urban innovation capacity.

#### 5.4.3 Urban wage levels

The LTCI can channel low-skilled workers into the care sector to meet the older adult care needs of high-skilled groups, thereby fostering a more efficient matching between low- and high-skilled labour. This improved labour matching contributes to raising urban wage levels, promoting high-quality economic development, and ultimately enhancing the overall consumption levels of urban households. We measure urban wage levels using hourly wages (*H_wage*) and average monthly income (*M_income*). To examine the urban wage levels channel through which the LTCI pilot increases household consumption, we re-estimate the baseline specification with these variables as dependent variables. The regression results presented in columns (5) and (6) of Table 7 show that the LTCI system significantly enhances urban wage levels, with increases in both hourly wages and average monthly income. This indicates that the wage levels of urban residents in pilot cities have increased as a result of the implementation of the LTCI system, thereby fostering high-quality urban economic development and exerting a positive impact on the consumption expenditure of urban households.

## 6 Scalability analysis

The extant literature has confirmed the significant role of regional spillovers of the impact of the resident income of the LTCI system in advancing common wealth (88). It is an irrefutable fact that an increase in resident income is a major factor in increasing household consumption. Therefore, this paper argues that there are spatial spillovers of the promotional effect of the LTCI system on urban household consumption. In particular, the local LTCI system pilot can increase urban household consumption in neighbouring areas.

The spatial spillover effect of LTCI on urban household consumption is primarily attributable to two factors, namely labour mobility transmission and spillover effects in medical service provision. On the one hand, the impact of LTCI can be transmitted to surrounding areas through labour mobility. When migrant workers enrol in LTCI in pilot cities, their family members remaining in their hometowns may receive indirect support through interregional policy linkages, reducing the financial burden of care and thereby enhancing their consumption capacity. Furthermore, LTCI’s stimulus to the healthcare and caregiving industries generates considerable labour demand (89), expediting the mobility of associated workers and the spillover of industries across regions. Consequently, this augments household incomes both within local areas and in proximate regions, resulting in heightened consumption expenditure through spatial spillovers. On the other hand, the regional aggregation of medical services engenders a multifaceted geographical spillover effect from the augmented provision of healthcare services under LTCI. Regions endowed with adequate healthcare resources manifest substantial spillover effects, establishing regional medical hubs that extend services to proximate areas (90). By fostering community-based and home-based care, LTCI mitigates demand pressure on healthcare resources and augments the local supply of medical services. This effect is further extended to adjacent regions through the regional clustering of healthcare services, thereby reducing the cost of accessing higher-quality medical care for residents in neighbouring areas. The decline in medical expenditures resulting from the policy alleviates the crowding-out effect on household consumer spending in areas near policy pilot zones, thereby generating a regional spillover effect of increased consumption.

To this end, this study employs the Spatial Autoregressive Model SAR, Equation 4 and the Spatial Durbin Model SDM, Equation 5 to examine the spatial spillover effect of LTCI on urban household consumption.

$$\begin{array}{c} \text{Consumptio}n_{\mathrm{it}}=\alpha_{1}+\rho_{1}W_{\mathrm{it}}\text{Consumptio}n_{\mathrm{it}}+\beta_{1}\text{Trea}t_{\mathrm{it}}\\ +\delta_{1}X_{\mathrm{it}}+\lambda_{t}+\mu_{i}+\varepsilon_{\mathrm{it}} \end{array}$$ (4)

$$\begin{array}{c} \text{Consumptio}n_{\mathrm{it}}=\alpha_{2}+\rho_{2}W_{\mathrm{it}}\text{Consumptio}n_{\mathrm{it}}+\beta_{2}\text{Trea}t_{\mathrm{it}}\\ +\theta_{1}W_{\mathrm{it}}\text{Trea}t_{\mathrm{it}}+\delta_{2}X_{\mathrm{it}}+\lambda_{t}+\theta_{2}X_{\mathrm{it}}+\mu_{i}+\varepsilon_{\mathrm{it}} \end{array}$$ (5)

where *W_it_* represents the spatial weight matrix, which is calculated as the inverse of the distance between cities, while *ρ* denotes the spatial correlation index of urban household consumption. The remaining variables are consistent with those defined in Equation 1. The regression results, presented in Table 8, show that regardless of whether the SAR or SDM model is applied, the *ρ* value is significantly positive. This indicates a significant positive spatial correlation in household consumption across different cities, supporting the argument of this study.

**Table 8:** Estimation results of the SAR and SDM models.

Variables	Dependent variable: urban household consumption (*lnUC*)	
	Equation 4: SAR model	Equation 5: SDM model
	(1)	(2)
*LT*	0.068** (2.46)	0.061** (2.40)
*Salary*	0.111** (2.04)	0.105** (2.02)
*Tax*	0.086*** (2.75)	0.068* (1.90)
*GDP_growth*	−0.002** (−2.23)	−0.002* (−1.93)
*Edu*	0.026** (1.73)	0.027* (1.78)
*Sec_ind_rate*	−0.146 (−1.36)	−0.166 (−1.57)
*Pop*	0.172 (0.11)	0.155 (1.45)
*Employees*	0.048* (1.90)	0.048** (1.99)
*Pollution*	−0.033 (−0.51)	−0.027 (−0.42)
*W_LT*		0.077 (1.61)
*W_Salary*		0.128* (1.86)
*W_Tax*		0.050 (1.09)
*W_GDP_growth*		−0.004 (−1.31)
*W_Edu*		0.006 (0.25)
*W_Sec_ind_rate*		0.055 (0.29)
*W_Pop*		0.180 (1.14)
*W_Employees*		−0.023 (−0.42)
*W_Pollution*		−0.054 (−0.39)
*ρ*	0.131*** (4.97)	0.114*** (4.21)
*θ^2^*	0.020*** (6.14)	0.019*** (6.11)
*City FE*	Yes	Yes
*Year FE*	Yes	Yes
*N*	1,832	1,832
*R^2^*	0.609	0.656

*** *p* < 0.01, ** *p* < 0.05, and * *p* < 0.10. T values are in parentheses.

Furthermore, we decompose the territorial and neighbouring effects of LTCI on household consumption using both models. Table 9 presents the regression results for the decomposition of direct and indirect effects. The consistently significant positive coefficients of the LTCI pilot policy in the spatial decomposition effects confirm that the implementation of LTCI in a given city leads to an increase in household consumption in neighbouring areas, demonstrating a significant positive spatial spillover effect. These findings provide empirical validation for the theoretical framework proposed in this study.

**Table 9:** The effect estimates of covariates in SAR and SDM models.

Variables	Dependent variable: urban household consumption (*lnUC*)	
	Equation 4: Spatial effect decomposition of SAR model	Equation 5: Spatial effect decomposition of SDM model
	(1)	(2)
Total effect		
*LT*	0.079** (2.44)	0.158*** (2.74)
*Salary*	0.128** (2.12)	0.258*** (2.64)
*Tax*	0.101*** (2.79)	0.131*** (2.78)
*GDP_growth*	−0.003** (−2.20)	−0.007* (−2.15)
*Edu*	0.030* (1.82)	0.039 (1.14)
*Sec_ind_rate*	−0.156 (−1.31)	−0.097 (−0.37)
*Pop*	0.200 (1.60)	0.374* (1.93)
*Employees*	0.054** (1.98)	0.030 (0.46)
*Pollution*	−0.041 (−0.57)	−0.101 (−0.62)
Direct effect		
*LT*	0.069** (2.44)	0.065** (2.50)
*Salary*	0.112** (2.10)	0.110** (2.16)
*Tax*	0.088*** (2.80)	0.071** (2.02)
*GDP_growth*	−0.002** (−2.19)	−0.002** (−2.03)
*Edu*	0.026* (1.82)	0.027* (1.89)
*Sec_ind_rate*	−0.135 (−1.31)	−0.153 (−1.51)
*Pop*	0.174 (1.60)	0.163 (1.48)
*Employees*	0.047** (2.00)	0.047** (2.07)
*Pollution*	−0.036 (−0.57)	−0.032 (−0.510)
Indirect effect		
*LT*	0.010** (2.18)	0.094* (1.74)
*Salary*	0.016** (1.99)	0.148** (2.00)
*Tax*	0.013** (2.32)	0.060 (1.23)
*GDP_growth*	0.000** (−2.02)	−0.005 (−1.46)
*Edu*	0.004* (1.67)	0.012 (0.40)
*Sec_ind_rate*	−0.020 (−1.20)	0.056 (0.25)
*Pop*	0.026 (1.51)	0.212 (1.25)
*Employees*	0.007* (1.68)	−0.016 (−0.27)
*Pollution*	−0.005 (−0.54)	−0.069 (−0.48)
*City FE*	Yes	Yes
*Year FE*	Yes	Yes
*N*	1,832	1,832
*R^2^*	0.609	0.656

*** *p* < 0.01, ** *p* < 0.05, and * *p* < 0.10. T values are in parentheses.

## 7 Research findings

Treating China’s LTCI pilot policy as a quasi-natural experiment, we explore its influence on urban household consumption and mechanisms through which it operates, leveraging panel data from 2011 to 2018 for 232 cities (prefecture-level and above). We find that the LTCI system significantly increased the level of urban household consumption expenditure with significant spatial spillover effects. The analysis of heterogeneity indicates that the LTCI pilot system has a greater effect on urban household consumption in regions experiencing net population inflow. Additionally, its impact is more pronounced in pilot cities with full coverage. Mechanism analysis indicates that increased provision of social healthcare services, enhanced urban innovation capacity, and rising wage levels for the labours are the main forces driving the growth of urban household consumption expenditure in the pilot LTCI system. LTCI, as a vital component of social security, serves to propel the economy towards higher-quality development by fulfilling the three fundamental functions of social security. Consequently, these measures have the potential to engender favourable outcomes for urban residents’ consumption expenditure. Firstly, the function of mitigating market failure enhances the supply of social healthcare and long-term care services, effectively increasing health capital and improving labour productivity. This establishes a robust health foundation for high-quality economic development while unlocking household consumption potential. Secondly, the risk-sharing function of the programme attracts highly skilled talent and incentivises family members to participate in the labour market by providing safeguards that reduce household care burdens and future expenditure uncertainties. This contributes to the enhancement of urban innovation capacity and knowledge creation dynamism, thereby driving industrial transformation and upgrading. Finally, the function of its resource allocation optimisation serves to enhance labour matching efficiency within the older people’s care services sector through institutionalised safeguards and employment opportunities. The resulting increase in wages is a key factor in stimulating urban residents’ consumption expenditure, thereby creating a positive feedback loop.

This conclusion is largely consistent with findings from countries such as Germany and Japan, where the establishment of LTCI systems has been shown to stimulate household consumption. However, the magnitude and transmission mechanisms of the effect in China differ markedly. Unlike the mature and uniform LTCI frameworks in advanced economies, China has adopted a multi-model pilot approach that allows policies to be tailored to the demographic, fiscal, and institutional characteristics of individual cities. This flexibility enhances the precision and adaptability of policy implementation. Moreover, the heterogeneity analysis reveals that the consumption-promoting effect of LTCI in areas with full coverage appears to be less pronounced in regions with partial coverage. Such differentiated pilot arrangements provide valuable empirical references for the nationwide rollout of LTCI, offering diversified policy pathways. From the perspective of household consumption alone, the evidence clearly indicates that a full coverage strategy outperforms a partial coverage model. This pilot-based and adaptive approach also distinguishes China’s policy experimentation from the more standardised systems observed in other countries. Overall, the findings of this study suggest that the consumption- and welfare-enhancing effects of LTCI are not confined to high-income countries. Rather, they hold significant implications for emerging economies undergoing rapid demographic ageing, where the development of inclusive and locally responsive care insurance systems may serve as an important mechanism for promoting domestic demand and improving social welfare.

## 8 Policy recommendations

Our findings yield critical policy implications. Firstly, it is recommended that China should accelerate the implementation of the LTCI system, expand the pilot, and improve the social security system. LTCI holds importance in promoting consumption expenditure, enhancing family welfare, and diversifying the long-term care market. Meanwhile, it also functions as a supplement to the existing social security system, underscores the necessity for China to proactively provide fiscal support and methodically expand the pilot scope of LTCI to optimise the system’s benefits. Secondly, to eliminate the intermediate barriers to the consumption demand released by LTCI, the government should increase investments in public medical service provision, offering more convenient healthcare conditions for urban residents, alleviating the shortage of medical resources, and meeting the medical needs of the population. *The 2019 Opinions on Promoting the Reform of the System and Mechanism for Social Mobility of Labor and Talent* relaxed urban settlement requirements for mid-sized cities (3–5 million population). This policy accelerated rural-to-urban migration, including older adult dependents, thereby elevating disability risks among ageing populations. Meanwhile, LTCI may substitute outpatient and inpatient demands, free public medical resources and lowering service prices to fully unlock the potential of urban household consumption and create a stable social environment, Policymakers should prioritise investments in public healthcare infrastructure. Thirdly, the LTCI system should be expanded to allow more urban residents to benefit from LTCI, balancing fairness and efficiency. The differences in the nature of employment act as a significant barrier to equal participation in medical insurance for urban populations. For basic medical insurance, the UEBMI enrolees’ contributions are shared between employers and individuals, offering a higher level of coverage and better benefits. Conversely, the URRBMI enrolees’ contributions are borne by individuals or households, resulting in lower coverage levels, higher deductibles, and lower reimbursement rates. At present, pilot cities are gradually covering both UEBMI enrolees and URRBMI enrolees, but disparities in care services and benefit standards persist. This has increased disability risks among older URRBMI enrolees, while their children shoulder a considerable psychological and financial burden. This, in turn, has a detrimental effect on the overall labour productivity of society. To optimise the beneficial impact of the LTCI system on urban household consumption, it is recommended that both UEBMI enrolees and URRBMI enrolees should enjoy equal access to LTCI benefits, in accordance with the principle of equal access to fundamental public services. The establishment of a fair and reasonably covered LTCI system is imperative to ensure equal participation and adequate protection for both categories of urban residents.

## Data Availability

The original contributions presented in the study are included in the article/supplementary material, further inquiries can be directed to the corresponding author.

## Footnotes

^1^Data sources: World Bank, Guotai Junan Securities Analysis and China Statistical Yearbook 2024.

^2^National Bureau of Statistics of China, July 15, 2022, Spokesperson of the National Bureau of Statistics Answers Reporters’ Questions on the Economic Performance in the First Half of 2022.

^3^World Health Organization (2021). Framework for countries to achieve an integrated continuum of long-term care.

^4^Central Committee of the Communist Party of China & State Council, 25 October 2016, *Outline of the “Healthy China 2030” Plan*.

^5^We select 13 pilot cities—Chengde, Changchun, Qiqihar, Shanghai, Nantong, Suzhou, Ningbo, Anqing, Shangrao, Qingdao, Guangzhou, Chongqing, and Chengdu—as the experimental group, while the remaining 219 cities that have not implemented the long-term care insurance system are treated as the control group. Cities such as Jingmen and Shihezi, where relevant variables are severely missing in the China Migrant Population Dynamic Monitoring Data and the China Urban Statistical Yearbook, are excluded from the analysis.

## Appendix

**Table A1:** Balance test.

Variables	Unmatched	Mean		%bias	%reduct	t-test	
	Matched	Treated	Control		|bias|	t	p > |t|
*Salary*	U	11.012	10.840	55.5	82.2	5.79	0.000
	M	10.956	10.925	9.9		0.64	0.524
*Tax*	U	8.794	8.133	69.5	81.6	7.39	0.000
	M	8.592	8.471	12.8		0.78	0.436
*GDP_growth*	U	8.924	8.988	−1.8	−388.2	−0.15	0.884
	M	8.844	8.534	8.8		0.68	0.499
*Edu*	U	4.688	4.602	16.0	89.9	1.48	0.140
	M	4.667	4.676	−1.6		−0.11	0.914
*Sec_ind_rate*	U	0.472	0.453	12.8	64.9	1.32	0.187
	M	0.478	0.485	−4.5		−0.3	0.764
*Pop*	U	6.719	5.843	142.5	98.5	12.63	0.000
	M	6.502	6.515	−2.1		−0.22	0.826
*Employees*	U	4.935	3.673	134.0	95.7	15.3	0.000
	M	4.565	4.510	5.8		0.42	0.676
*Pollution*	U	0.148	0.075	64.2	78.2	9.03	0.000
	M	0.103	0.119	−14.0		−1.09	0.279
